# Combined Penetrating Keratoplasty and Vitrectomy: Long-Term Follow-Up Results

**DOI:** 10.3390/jcm13237468

**Published:** 2024-12-08

**Authors:** Orit Vidne-Hay, Amir Alhalel, Irina S. Barequet

**Affiliations:** 1 Goldschleger Eye Institute, Sheba Medical Center, 5262000 Ramat Gan, Israelibarequet@yahoo.com (I.S.B.); 2 Faculty of Medical & Health Sciences, Tel Aviv University, 52621 Tel Aviv, Israel

**Keywords:** penetrating keratoplasty, keratoprosthesis, pars plana vitrectomy, corneal graft, silicone oil

## Abstract

**Purpose**: To assess the long-term outcomes of combined penetrating keratoplasty (PKP) and pars plana vitrectomy (PPV). **Methods**: A retrospective review of eyes that underwent combined surgery followed for 12 months or longer. Demographic data, indications for surgery, and pre-/post-surgical eye examinations were retrieved. **Results**: Thirteen consecutive eyes (13 patients) were enrolled. The mean age was 51.5 ± 20.5 years, and the mean follow-up time was 67 ± 36.9 months. All cases had severe corneal opacity. Indications for surgery included retinal detachment (76.9%), dropped lens (7.7%), dropped intraocular lens (7.7%), and endophthalmitis with corneal abscess (7.7%). Visual acuity improved in 46.1% of the cases, though in most cases visual acuity remained low, and decreased in 23% of the cases. In 23% of the cases (3 eyes), ambulatory vision was achieved for more than 12 months and in 15.4% for three years. Silicone oil tamponade was used in all cases of retinal detachment (10 eyes). Of these eyes, at the final follow-up, four eyes were attached, two eyes were partially attached, and corneal opacity prevented retinal visualization in three eyes. One eye was eviscerated and one eye developed phthisis. Postoperatively, 61.5% of the cases underwent repeated PKP for graft decompensation. At the final visit, graft failure was observed in 75% of the cases. **Conclusions**: The long-term follow-up of eyes that underwent combined PKP and PPV supports this technique in complex cases for eye and vision preservation. The main problem after combined surgery is the long-term survival of the corneal graft which may require repeated PKP surgeries. With this approach, in 23% of the cases, ambulatory vision was maintained for more than 12 months.

## 1. Introduction

Eyes with a coexisting corneal and vitreoretinal pathology present a challenge for treatment and follow-up. In eyes with severe corneal opacity that require vitrectomy for retinal detachment, dropped lens or intraocular lens (IOL), endophthalmitis, or trauma, vitrectomy combined with keratoplasty is sometimes the preferred surgical procedure. In these cases, a temporary keratoprosthesis (TKP) enables adequate visualization of the posterior chamber during vitrectomy. After performing the vitrectomy, the TKP is replaced by a corneal graft [1]. Landers temporary keratoprosthesis was introduced in 1981 [2]. It was 7.2 mm in diameter with an optical cylinder of 5 mm and was made of polymethyl methacrylate (PMMA). The modified Landers third-generation wide-field TKP is a PMMA device with a 1 mm cylinder protruding from the anterior chamber, and a corneal surface diameter of 15.5 mm [3]. The Landers keratoprosthesis is durable, maintains intraocular pressure during indentation, and enables a good view of the peripheral retina [4,5,6].

Several retrospective studies have described the use of temporary keratoprosthesis in combined keratoplasty and vitrectomy for many indications and with a wide range of anatomical and functional results [7,8,9,10,11,12,13,14]. In most studies, the sample size is small, and the follow-up time is relatively short. By contrast, the current study describes the long-term results of cases that underwent combined penetrating keratoplasty and vitrectomy with the Landers keratoprosthesis.

## 2. Material and Methods

A retrospective study of 13 consecutive eyes of 13 patients was conducted. All cases underwent penetrating keratoplasty and vitrectomy with the Landers temporary keratoprosthesis at the Sheba Medical Center, Israel. The Institutional Review Board of Sheba Medical Center approved the study (Approval code 4537-17-SCM, 29 November 2017). Informed consent was waived.

The patients’ data were retrieved from their medical records from January 2005 to December 2017. Data were abstracted from surgical reports and inpatient and outpatient clinical charts. Only eyes with a follow-up of more than 1 year were included. Demographic information was obtained (age, sex, study eye, etiology of the ocular problem, and the indication for the combined surgery), as well as pre- and post-surgical eye examinations, including best-corrected Snellen visual acuity (VA), intraocular pressure (IOP), and corneal, lens, and retinal status. Post-surgery, the corneal graft status was defined as clear (1), mild opacity (2, good visualization of the retina), moderate opacity (3, optic nerve, retinal major vessels, and areas of detachment could be visualized), severe opacity (4, the retina could not be visualized), or/and graft decompensation. The retinal status was noted at baseline and final follow-up. The retinal status was defined as completely attached (1), mostly attached (2, area of attached retina > 75%), partially attached (3, area of attached retina = 74–50%), mostly detached (4, area of attached retina = 49–25%), total retinal detachment (5), and no visualization (6). In eyes with silicone oil tamponade, the presence of silicone oil in the anterior chamber, the presence of emulsification, and the neovascularization of the iris were recorded. Data on the length of follow-up, previous surgeries, additional surgeries, and complications, including silicone emulsification, corneal graft failure, and glaucoma were also obtained. In eyes with glaucoma, the number of glaucoma medications and any surgical procedures performed were noted.

Both corneal surgeons and vitreoretinal surgeons performed the surgical procedures in this cohort. All surgeries were carried out under general anesthesia. The surgical procedure was performed as previously described by Ikeda et al. [1]. Initially, an infusion port was inserted at the pars plana, and two more ports were prepared at the pars plana. Then, a Flieringa ring was sutured to the sclera with 5.0 silk sutures. A corneal window was made using a corneal trephine of 7.25 mm in diameter. The 7.2 mm Landers keratoprosthesis was inserted in the corneal window and sutured to the corneal rim and sclera at 4–6 sites using 7–0 silk sutures.

After the insertion of the corneal prosthesis, a standard 20/23 Gauge 3 port pars plana vitrectomy was performed. In cases with a detached retina, the retina was reattached with perfluorocarbon liquid, followed by a 360-degree laser, PFC–air exchange, and silicone oil injection (5000 cs silicone oil). After vitrectomy, the keratoprosthesis was removed, and a corneal graft, 0.25 mm larger than the corneal window, was sutured with interrupted 10/0 nylon sutures. Depending on the lens, intraocular lens, and retinal pathology, additional procedures were performed during surgery, including open-sky intraocular lens removal (two eyes), removal of a dropped lens with the vitrectomy (one eye), and 360-degree buckling (one eye).

Postoperatively, all eyes were treated for 1 month with topical antibiotics and steroids (Dexamethasone as sodium phosphate 0.1%, Fischer Pharmaceuticals LTD) for the long-term. Steroids were gradually tapered down and adjusted according to the corneal graft clarity, inflammation of the eye, and intraocular pressure.

## 3. Results

Eyes from ten males and three females were included in the study. The right eye was affected in five cases and the left eye in eight cases. The mean age at presentation was 51.5 ± 20.5 years (range: 19–81 years), and the mean follow-up time was 67 ± 36.9 months (range: 15–113 months). Table 1 presents a summary of these cases.

The indications for combined surgery included the presence of severe corneal opacification with retinal detachment in 76.9% of the cases (10 eyes), dropped lens (1 eye), dropped IOL (1 eye), and endophthalmitis secondary to a corneal abscess (1 eye).

A history of ocular trauma was present in 61.5% of the cases (eight eyes). Of these cases, seven had a history of perforating trauma, and one eye had a history of blunt trauma.

In 92.3% of cases (12 eyes), at least one ocular surgery was performed before the combined penetrating keratoplasty and vitrectomy. In one eye, the combined penetrating keratoplasty and vitrectomy was the first surgery. Previous surgeries included glaucoma surgery in three eyes (23% cases 3, 4, and 12 in Table 1), corneal transplantation in six eyes (46.2%), cataract surgery in nine eyes (69.2%), and posterior vitrectomy in three eyes (23%).

All the eyes had corneal opacification at presentation which did not enable proper visualization of the posterior chamber. At the final follow-up, 75% of the cases (nine eyes) had severe corneal graft opacification. In 25% of the cases (three eyes), the corneal graft was clear, but only in one eye; the clear graft was from the primary combined penetrating keratoplasty and vitrectomy.

At presentation, 30.7% of the cases (four eyes) were phakic. These eyes underwent cataract surgery during the primary combined penetrating keratoplasty and vitrectomy. At the final follow-up, 83.3% of cases (10 eyes) were aphakic, and 16.7% of cases (2 eyes) had a PCIOL (Table 1). In all aphakic eyes at the final follow-up, it was considered that an IOL implantation would not improve visual acuity.

The retina was detached in 76.9% of cases (10 eyes) at presentation and attached in 23% of cases (3 eyes). Silicone oil tamponade was used in all cases with retinal detachment. At the final follow-up, four eyes were attached, two eyes were partially attached, and corneal opacity prevented retinal visualization in six eyes. One eye was eviscerated.

In 46.2% of the cases (six eyes), visual acuity had improved by the final follow-up but remained low in almost all these eyes (Table 1). In 23% of the cases (three eyes), visual acuity decreased, whereas in 30.8% of the cases (four eyes), it did not change. One eye achieved an ambulatory final visual acuity of 20/125.

Table 2 presents the visual acuity results for the first 3 years of the follow-up. Ambulatory vision (defined as visual acuity better than 20/400) was achieved in 23% of the cases (three eyes) at the 1-year postoperative follow-up and in 15.4% of cases (two eyes) at the 3-year postoperative follow-up. In the latter, visual acuity decreased in the fourth year due to corneal graft decompensation. Crucially, these 2 cases underwent severe perforating trauma, were aphakic, and the silicone oil was in contact with the corneal graft. In one eye, the retina was attached. In the second eye, the retina was 75% attached. The visual loss occurred secondary to corneal decompensation.

In 92.3% of the cases (12 eyes), additional surgeries were required during follow-up, including three-port pars plana vitrectomy, silicone oil exchange, silicone oil removal, penetrating keratoplasty, and tarsorrhaphy. The mean number of additional surgical procedures was 3 ± 2.83 (range 0–9). Only one eye did not undergo any additional surgery. This eye developed phthisis, and no surgery was recommended. Repeated penetration keratoplasty was required in 61.5% of the cases (eight eyes). Corneal graft decompensation occurred during the first postoperative year. Four cases underwent more than one penetrating keratoplasty (range 2–5), and 53.8% of the cases (seven eyes) underwent recurrent three-port pars plana vitrectomy for recurrent retinal detachment or epiretinal membrane peeling. Silicone oil removal was performed in 40% of the cases (four eyes).

The silicone oil was in contact with the corneal graft in 70% of cases (seven eyes) and with silicone oil tamponade throughout the follow-up. Out of the five eyes with silicone oil at the final follow-up (cases 1–5), silicone oil emulsification was recorded in three eyes. In two eyes, emulsification could not be determined due to severe corneal opacity. One eye developed phthisis but silicone oil emulsification was seen in the anterior chamber.

Glaucoma developed in 15.4% of the cases (two eyes) and was controlled with topical treatment (one–two types of topical drops).

## 4. Discussion

This series reports the long-term follow-up of 13 eyes with severe corneal opacity and posterior segment pathology that underwent combined penetrating keratoplasty and vitrectomy using Landers temporary keratoprosthesis.

Combined penetrating keratoplasty and vitrectomy has been described in the literature, along with the temporary keratoprosthesis [1,2,15]. This procedure is a viable option for preserving the eye and vision in cases with combined anterior and posterior chamber pathology. A clear corneal graft allows for the follow-up of the retina after surgery. Endoscopic vitrectomy is another possible surgical procedure in these complex cases, followed by penetrating keratoplasty in a separate setting for cases in which the cornea remains opacified. Chun et al. [16] obtained comparable short-term anatomic and functional results when comparing the two procedures. However, in eyes with silicone oil, retinal follow-up is compromised since ultrasound cannot adequately assess the retinal status.

In the current series, 69.2% of the cases achieved an improvement or stabilization of vision, though in most cases visual acuity remained low. This finding is consistent with previous reports [3,10]. However, in the present study, 23% of cases achieved ambulatory vision by the end of the first postoperative year, and 15.4% in the third postoperative year. Since these are significantly complicated cases with poor prognosis, the ability to maintain vision for several years is of great importance.

The main cause of visual deterioration was graft failure. In cases where the retina could be visualized during follow-up, it was attached or mostly attached, but vision deteriorated due to progressive clouding of the corneal graft. Thus, the main problem associated with this type of combined surgery is the long-term survival of the corneal graft.

Nowomiejska et al. [3] reported a 1-year follow-up on combined surgery for ocular trauma and found that although the retina was attached in most cases, the corneal graft survived in only 25% of the eyes, and final visual acuities were low. Lee et al. [12] reported combined surgery results in cases with corneal pathology and vitreoretinal diseases such as endophthalmitis, posterior uveitis, vitreous opacity, and hemorrhage. Following surgery, 27.3% of the corneal grafts survived. The graft survival rate was a function of the existence of active inflammation in the cornea pre-surgery. The mean graft survival time was 391 days. Dong et al. [17] reported on 107 trauma-related eyes that underwent combined surgery. The mean follow-up time was 25 months, and the overall corneal graft failure rate was 31.8%, which is lower than in other studies. They noted that a higher success rate was achieved in cases with early surgical intervention (within 1 month of ocular injury = 81.9%) as opposed to late surgical intervention (1 month or more after injury = 54.3%). Khouri et al. [18] documented a series of 24 eyes that underwent combined surgery with a mean follow-up of 36 months. At the final follow-up, the corneal graft was clear in 18 eyes (79%), whereas 5 eyes (21%) had graft failure. Yu et al. [14] reported an overall rate of graft failure in 60.9% of eyes. In a multivariable analysis, they found that ocular trauma and intraocular silicone oil both significantly increased the graft failure risk by more than 5-fold. After excluding the cases of trauma and silicone oil, the overall graft failure rate decreased to 33.3%.

In the current cohort, 75% of the eyes had developed graft failure by the final follow-up, and 61.5% of the eyes underwent additional keratoplasty to preserve visual acuity. These percentages are comparable with most reports. The longer follow-up in our study can partially explain the low survival rate of the corneal graft. The high rate of cases with ocular trauma and intraocular silicone oil can explain the high failure rate as shown by Yu et al. Dong et al. [17] reported a higher rate of graft survival. This difference can be explained by the fact that in our series, most eyes had a complex previous ocular history and underwent at least one surgery before the combined penetrating keratoplasty and vitrectomy; 46.2% of eyes underwent previous corneal transplantation. Moreover, most cases underwent surgery more than one month after the trauma. The lower rate of silicone oil tamponade used by Dong et al. can also explain their lower rate of graft failure.

Corneal graft failure can also occur as a result of prolonged inflammation following surgery in these highly complex cases that affects the corneal graft’s viability, despite continuous corticosteroid treatment, as suggested by Lee et al. [12]. Another explanation has to do with the fact that in 76.9% of the cases, silicone oil was injected and in 70% of the cases, the oil was in contact with the corneal graft. The silicone oil could only be removed one year postoperatively in 40% of the cases as opposed to Dong et al. [17]. Silicone oil was required in these cases for the long-term tamponade given the complicated retinal detachment. Silicone oil is thought to reduce the rate of PVR development, but in cases where it is in direct contact with the cornea, it can promote corneal graft failure, as shown by Roters et al. [11] and Yu et al. [14]. In our cases, silicone retention sutures were not used. It could be that in future cases, especially in trauma eyes, aphakic eyes, and eyes with aniridia, silicone retention sutures should be considered [19].

The corneal pathology in our series included three eyes with significant corneal melting and one eye with a severe corneal graft infiltrate at presentation. In two eyes, vision improved slightly, whereas in two eyes vision did not improve. Although combined surgery is essential in such cases, the literature reflects the poor outcome of these types of corneal pathologies. Dave et al. [8] reported on combined surgery for the treatment of infective endophthalmitis. Their report included eyes with a corneal ulcer (69.75%) and eyes with a corneal graft infection (18.65%). Anatomical failure occurred in 34.9% of the cases (15 eyes), of which 9 eyes developed phthisis, and 6 of the phthisic eyes were eviscerated. Dong et al. [17] reported eye salvage in 86% of the cases with combined surgery, but in their series, only 4.6% were operated on for endophthalmitis secondary to keratitis. Tanaka et al. [9] reported results on 15 patients with rejected corneal grafts who underwent combined surgery for proliferative vitreoretinopathy (PVR) or fungal endophthalmitis. Postoperatively, 46% of the eyes had a clear corneal graft at 6 months, whereas 40% of the eyes had low postoperative vision and eventually developed phthisis.

A possible option to be considered for cases with repeated graft failures is the implantation of a Boston type I keratoprosthesis, but the few long-term follow-up studies that have been published report limited anatomical and visual improvement [20,21].

The current study is limited by its small sample size, the heterogeneity of the cases, and the retrospective evaluation of long-term data. Because of the small number of cases, statistical analysis is limited. In addition, surgeries were performed using both 20-gauge and 23-gauge pars plana vitrectomy. The 20-gauge vitrectomy requires port marking and the suturing of the insertion site, whereas the 23/25-gauge vitrectomy, which uses trocar insertion, has the advantages of a faster procedure and does not require suturing. However, many of our surgeries were performed before the development of the 25/23-gauge system. Although vitrectomy techniques have evolved over the last ten years, the indications for combined surgery, the postoperative follow-up, and treatment strategies remain similar, thus authorizing the evaluation of this unique group of eyes. In the future, multicenter studies can improve our understanding and management of these rare and challenging cases.

Overall, the long-term follow-up in this series of combined keratoplasty and pars plana vitrectomy supports the salvage of most eyes and the preservation of vision. This technique allows for optimal visualization during pars plana vitrectomy and proper post-surgery retinal follow-up in extremely complicated cases. Roughly one-quarter (23%) of the cases maintained ambulatory vision for more than 12 months.

## Figures and Tables

**Table 1:** Patient data.

			Initial Ocular Exam				Final Ocular Exam						
	Age (years)	Diagnosis	VA	Cornea	Lens	Retina	VA	Cornea	Lens	Retina	F/U (months)	Complications	Remarks
1	46	Perforation- blunt trauma	CF	4 (CG)	Aphakia	3 (SO)	CF	4(+CGR)	Aphakia	1	87	SO emulsification	
2	37	Perforation- blunt trauma	HM	4 (CG)	Aphakia	3 + VH	CF	3	Aphakia	1	24	SO emulsification	
3	77	CGR + RD	CF	4+ CGR	PCIOL	3	NLP	4	PCIOL	6	55	Glaucoma+ Phthisis SO emulsification in AC	Pre-surgery glaucoma S/P trabeculectomy
4	68	CGM + RD + endophthalmitis	LP	4+ CGM	ACIOL	3	LP	4(+CGR)	Aphakia	6	113	Glaucoma	Pre-surgery glaucoma S/P trabeculectomy
5	29	Perforation due to explosion	LP	4	Traumatic cataract	4	NLP	4	Aphakia	6	105		
6	56	CG abscess + RD S/P perforation due explosion	HM	4 (CG abscess)	Aphakia	3	CF	4	Aphakia	1 (Per US)	57	Glaucoma	
7	42	Perforation (from explosion)	HM	4	Traumatic cataract	4	HM	4	Aphakia	3 (Per US)	112		
8	69	Leukoma adherence + RD	HM	4	NS + 2	3					60	Evisceration	
9	74	Corneal melting + dropped IOL	HM	4	Dropped PCIOL	1	CF	3	Aphakia	1	36		
10	37	CGR + RD S/P perforation due to explosion	HM	4 + CGR	PCIOL	3	CF	1	Aphakia	2	124		
11	81	Corneal melting + endophthalmitis	LP	4	PCIOL	1	CF	1	PCIOL	1	45		
12	15	Corneal melting + dropped lens S/P blunt trauma	HM	4	Dropped lens	1	HM	4	Aphakia	6	39	Glaucoma	Peters anomaly Pre-surgery glaucoma S/P Ahmed valve implantation
13	34	Corneal scar + RD S/P perforation	CF	4	PCIOL	3	20/125	1	Aphakia	1	15	Glaucoma	

VA = Visual acuity, HM = Hand motion, LP = Light perception, CF = Counting fingers, NLP = No light perception, CG = Corneal graft, CGR = Corneal graft rejection, CGM = Corneal graft melting, ACIOL = Anterior chamber intraocular lens, PCIOL = Posterior chamber intraocular lens, NS = Nuclear sclerosis, RD = Retinal detachment, IOL = Intraocular lens, VH = Vitreous hemorrhage, AC = anterior chamber, SO = Silicone oil, Per US = Per ultrasound. Corneal graft grading: 1—clear, 2—mild opacity (good visualization of the retina), 3—moderate opacity (optic nerve, retinal major vessels, and areas of detachment could be visualized), 4—severe opacity (retina could not be visualized), or/and graft decompensation. The retinal status is defined as completely attached (1), mostly attached (2, area of attached retina > 75%), partially attached (3, area of attached retina = 74–50%), mostly detached (4, area of attached retina = 49–25%), total retinal detachment (5), and no visualization (6).

**Table 2:** Changes in visual acuity over the course of the follow-up.

Patient	Initial (Pre-Surgery) VA	1 Year Post-Surgery	2 Years Post-Surgery	3 Years Post-Surgery	Final VA	Best VA Post-Surgery	FU Time (Months)
1	CF	CF	CF	CF	CF	CF	87
2	HM	CF	CF		CF	CF	24
3	CF	HM	HM	LP	NLP	HM	55
4	LP	HM	HM	LP	LP	LP	113
5	LP	CF	CF	CF	NLP	CF	105
6	HM	20/125	20/200	20/200	CF	20/125	57
7	HM	HM	LP	HM	HM	LP	112
8	HM	HM	HM	HM	NLP	HM	60
9	HM	CF	CF	CF	CF	CF	36
10	HM	20/100	20/100	20/100	CF	20/100	124
11	LP	HM	HM	CF	CF	CF	45
12	HM	HM	HM	HM	HM	HM	39
13	CF	20/125			20/125	20/125	15

VA = Visual acuity, FU = Follow-up, LP = Light perception, HM = Hand motion, CF = Counting fingers.

## Data Availability

The raw data supporting the conclusions of this article will be made available by the authors on request.
